# Stress before Puberty Exerts a Sex- and Age-Related Impact on Auditory and Contextual Fear Conditioning in the Rat

**DOI:** 10.1155/2007/71203

**Published:** 2007-06-24

**Authors:** Maria Toledo-Rodriguez, Carmen Sandi

**Affiliations:** Brain Mind Institute, Ecole Polytechnique Federale de Lausanne (EPFL), 1015 Lausanne, Switzerland

## Abstract

Adolescence is a period of major physical, hormonal, and psychological changes. It is also characterized by a significant increase in the incidence of psychopathologies and this increase is gender-specific. Stress during adolescence is associated with the development of psychiatric disorders later in life. In this study, we evaluated the impact of psychogenic stress (exposure to predator odor followed by placement on an elevated platform) experienced before puberty (days 28–30) on fear memories and hormonal response of male and female rats during adolescence and early adulthood. Stress before puberty impacted in a sex- and age-specific way on the responses to auditory and contextual fear conditioning in adolescence and adulthood: (a) increased conditioned fear to the tone in males during adolescence but not during adulthood; (b) impaired extinction to the tone in adult males; and (c) reduced freezing responses to the context in adolescent females. Stress before puberty did not influence the corticosterone levels 30 minutes after an additional stressor given in adulthood. These results indicate that stress experienced prior to puberty can exert a sex-related differential impact on fear-related behaviors displayed by individuals during late adolescence and early adulthood.

## 1. INTRODUCTION

Adolescence is a critical developmental period characterized by major physical, hormonal, and psychological changes. In humans, adolescence is also accompanied by sharp and gender-related increases in morbidity and susceptibility to psychopathologies [1, 2]. During adolescence, the incidence of depression, post-traumatic stress disorder (PTSD), anxiety, and eating disorders specifically increase among girls [3], while boys display higher tendencies to develop aggression, substance abuse, and novelty-seeking behaviors [4].

In humans, adolescence is characterized by major developmental changes in the brain and the hypothalamicpituitary- adrenal axis (HPA axis). Each system matures at a different pace, with the HPA axis maturing before the brain pathways that regulate emotion, cognition, and learning (prefrontal cortex [PFC], hippocampus, amygdala, and ventral striatum) [5, 6]. These dynamic changes in stressresponsive regions are theorized to contribute to the unique behavior of adolescents and their increased susceptibility to develop psychopathologies [1, 7, 8]. In humans, stress is a known risk factor leading to the development of psychopathological alterations [9] and empirical evidence indicates that stressful experiences during adolescence are associated with the development of psychiatric disorders later in life [2, 10, 11].

In the rat, adolescence is considered to last from around P21–28 to P55–60. One widely accepted classification (as reviewed by McCormick and Mathews [12]) divides adolescence in 3 periods: prepubescence or early adolescence (from weaning, usually P21 to P34); pubescence or midadolescence (from P34 to P46); and postpubescence or late adolescence (from P46 to P60). For Wistar rats, the onset of puberty for females (vaginal opening) occurs around P34–36 while for males (balanopreputial separation) occurs around P40–42 [13]. As in humans, adolescence in the rat is also characterized by major developmental changes of the brain and HPA axis (see [14], for review see [15]).

Recently, different laboratories have studied, in the rat, the impact of stress experienced during adolescence upon adult behavior [16–19], endocrine responses [20–23], neuronal and brain morphology [23, 24], and drug sensitization [21, 22, 25]. Stressors were applied either before puberty [16–20], around puberty [21, 22], or during the entire adolescence [23–25]. With the exception of a few reports that include males and females [21, 22, 26], the majority of these studies focused only on males.

There is an increasing body of evidence indicating that the learning and memory processes of adult male and female rats are differentially impacted by stress [27–30]. Under nonstress conditions, males outperform females in tasks involving spatial components, such as the radial arm maze [31], object placement [32], the Y maze [33], and the Morris water maze [34]. This pattern is reversed when the animals are submitted to stress in adulthood. Chronic stress improves spatial memory in adult females, while it impairs performance in adult males [32–35] when compared to their respective controls. Interestingly, Shors and colleagues found the opposite for the eye blink conditioning task. In adulthood, females outperformed males under non-stress conditions. However, exposure to a short stressful experience (30 minutes restrain paired with tail shocks) impaired performance in females, whereas improved performance in males [26]. The same study showed that eye blink conditioning was not affected when stress was performs prior to puberty. Nevertheless, it should be noted that this study focused on the short-term (48 hours) effects of stress.

In humans, a large body of empirical evidence indicates that stress experienced during early life is associated with an increase in the prevalence of psychopathologies such as depression, PTSD, and anxiety disorders [36]. In the rat, there are however only a handful of studies investigating the priming effects of adolescent stress upon the subsequent impact of stressful experiences in adulthood [17–19]. These studies showed that stress prior to puberty onset altered the behavioral consequences to a second stress in adulthood: it increased the susceptibility to develop learned helplessness in the shuttle box [18] while it increased the performance in the Morris water maze [17]. In rodents the fear conditioning task is commonly used to model the development of fear memories as well as some of the symptoms of PTSD. During fear conditioning, the animals learn the association between a context or cue (usually a tone) and an electric shock. This task has been widely used to study the cellular and molecular basis of acquisition, maintenance and extinction of aversive memories [37]. We are not aware of any study that focused on the priming effects of adolescent stress upon the acquisition, storage, and expression of fear memories in the rat.

The goal of this study was to evaluate the impact of psychogenic stress prior to puberty onset upon the acquisition, maintenance, and extinction of fear memories at both midadolescence and adulthood. Since we hypothesized that stress would have a differential impact in male and female rats, a second aim of the study was to evaluate the impact of stress on fear memories in each sex, and then to compare potential differential responses between them. Our stress protocol was applied at the same time period as the juvenile stress model developed in males by the group of Richter-Levin [17], although we used different stressors. Briefly, we exposed male and female rats to three consecutive days of psychogenic stress (exposure to predator odor followed by placement on an elevated platform, P28–P30) and then, after a first characterization of their general behavior during midadolescence (P39-P40), they were submitted to fear conditioning protocols both in midadolescence (P41-42) and early adulthood (P83–85), with extinction processes being explored in adulthood (P86-87). Finally, the corticosterone response 30 minutes after an acute (heterotypic) stressor in adulthood was examined.

## 2. MATERIALS AND METHODS

### 2.1. Subjects

Subjects were the offspring of rats purchased from (Charles River Laboratories, France). Nineteen male and twelve female Wistar Han rats from different litters were weaned at postnatal day 21. Rats from different litters were mixed, placing equivalent numbers of animals from each litter in stress and control groups. Rats were housed in same sex cages (3 or 4 per cage) in standard plastic cages on a 12-hours light-dark cycle (light on at 7:00 AM). Food and water were available ad libitum. All the procedures described were conducted in conformity with the Swiss National Institutional Guidelines on Animal Experimentation, and approved by the Swiss Cantonal Veterinary Office Committee for Animal Experimentation.

### 2.2. Stress procedure before puberty onset

At the age of P28–P30 (3 consecutive days) 16 rats (10 males and 6 females) were submitted to a protocol of stress before puberty modified from Avital and Richter-Levin [17]. The timing of the stress was random (unpredictable for the animal) but always during the light period. Stress was composed of 2 subsequent stressors: exposure to trimethylthiazoline (TMT) odor (Phero Tech Inc. Delta, Canada) during 25 minutes in a closed box 38 × 27.5 × 31 cm dimensions under bright light (200–250lux) containing a cloth impregnated with 10mg TMT; followed by exposure to an elevated platform (of 12 × 12 cm located 94.5cm from the ground) during 25 minutes under direct bright light (500–550lux). Control rats remained undisturbed in their home cages.

### 2.3. Activity box and reactivity to novelty

One week after stress all rats (stressed and control) were handled during 2 minutes daily for 3 days before testing their spontaneous locomotor activity in an activity box. The activity box consisted in a rectangular arena (of 37 × 57 cm) divided in 9 zones of identical size. The test was started by placing the animals in the center of the arena and lasted 10 minutes. Right after, a novel object (green glass bottle of 33cc) was placed in the center of the arena and the reactivity of the animal to the novelty was measured for 5 minutes. The locomotor activity was monitored by a video camera, mounted on the ceiling and a computerized tracking system (Ethovision 1.90, Noldus IT,Wageningen, The Netherlands), recorded the total distance moved and speed, percentage of time spent in each zone, and latency to enter the center. The floor of the arena was washed after each testing with 0.1% acetic acid solution to remove odors left by previous subjects.

The test was performed simultaneously to all the animals in the cage by using 3 or 4 adjacent arenas.

### 2.4. Elevated plus maze

Twenty four hours after the activity box and reactivity to novelty tests, anxiety-like behavior in stressed and control rats was evaluated using the elevated plus maze (EPM) test. The EPM consisted of two opposing open arms (45 × 10 cm) and two closed arms (45 × 10 × 50 cm) that extend from a central platform (10 × 10 cm) elevated 65cm above the floor. The rats were placed individually on the central platform facing a closed arm and were allowed to freely explore the maze for 5 minutes. The behavior of each rat was monitored using a video camera, and the movements of the rats were automatically registered and analyzed with a computerized tracking system (Ethovision 1.90, Noldus IT, The Netherlands). Entry into an arm was defined as entry of all four paws into the arm.We recorded total distance moved, speed, time spent in the open and closed arms, number of times the animal entered each type of arms, latency before entering an open arm and number of defecations.

### 2.5. Fear conditioning

**Figure 1:**
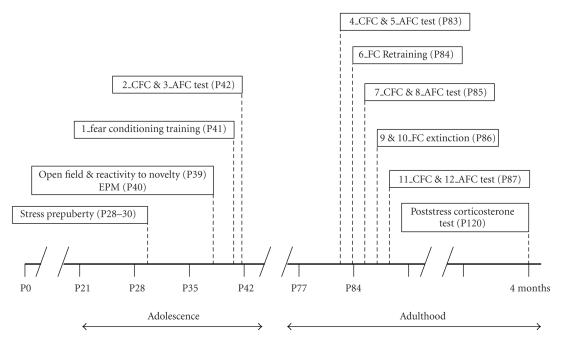
Stress and behavioral test procedures. “P” indicates the postnatal day. CFC: contextual fear conditioning test; AFC: auditory fear conditioning test.

Training and testing took place in three identical rodent observation cages (30 × 37 × 25 cm) placed into a soundattenuating chamber, illuminated with a 20W bulb. The sidewalls of the observation cage were constructed of stainless steel and the door of clear Plexiglass. The floor consisted of 20 steel rods wired to a shock source and solid-state scrambler for the delivery of foot shock unconditioned stimuli (US). Ventilation fans provided a background noise of 68 dB (the whole Fear System was acquired from Panlab, Spain). Animals were transported from the colony room to the adjacent fear conditioning room where training and testing took place. After each testing session, animals were returned to their home cages. The animals' behavior was recorded and later scored with an in-house-made behavior observation software (Clicker v1.13) by an observer blind to the treatment or sex. Indicator of fear was freezing, which is defined as behavioral immobility except for respiration movements for at least 2 seconds. Freezing times were automatically transformed to percentage freezing values. We measured percentage of time freezing, average length of the freezing events, number of freezing events, and latency to freeze. Below, there is a detailed description of the experimental schedule used (see Figure 1 for a schematic representation of the protocol).

*1_Auditory fear conditioning (AFC) training in adolescence*

Eleven days after the termination of the stress procedure and the day after the EPM test, stressed and control rats (age P41) were submitted to AFC training. Rats were exposed to Context A (black walls of smooth texture, steel grid floor, cleaned with 2% ethanol) during 160 seconds, followed by three presentations of tone-shock pairings in which the tone (20 seconds) coterminates with a foot shock (0.4 mA, 1 second). The intertone interval was 40 seconds and the conditioning session lasted 5 minutes and 30 seconds in total. Behavioral responses prior to, during, and immediately following the tone and shock pairings were measured.

*2_Contextual fear conditioning (CFC) test in adolescence*

One day after AFC training (i.e., at age P42), animals were exposed to the same context (Context A) where the fear conditioning training took place and left undisturbed for 8 minutes. Behavioral responses to the conditioned context were measured.

*3_AFC test in adolescence*

Two to four hours after CFC test at age P42, animals were introduced into a box displaying a new context, Context B (green walls of rough texture, grey plastic floor covered with flocks, cleaned with 4% chlorine), for 8 minutes during which they were exposed to conditioned tone during the last 5 minutes. Behavioral responses to the conditioned tone were measured.

*4_CFC memory test in adulthood*

Six weeks after fear conditioning training (i.e., at age P83), animals were exposed to the conditioned context (Context A) and left undisturbed for 8 minutes. Behavioral responses to the conditioned context were measured.

*5_AFC memory test in adulthood*

Two to four hours after CFC test at age P83, animals were placed into Context B for 8 minutes, where they were exposed to the conditioning tone during the entire last 5 minutes of the test. Behavioral responses to the conditioned tone were measured.

*6_AFC retraining in adulthood*

At age P84, all animals were submitted to a retraining auditory fear conditioning session, similar to the one performed during adolescence (number and duration of tone and shocks; Context A; see 1_Auditory fear conditioning (AFC) training in adolescence). Behavioral responses prior to, during, and immediately following the tone and shock pairings were measured.

*7_CFC memory test after retraining in adulthood*

The day after adult retraining, animals (aged P85) were submitted to a context memory test performed as described in 4 CFC memory test in adulthood (i.e., exposure to the conditioned Context A during 8 minutes).

*8_AFC memory test after retraining in adulthood*

Two to four hours after CFC test at age P85, animals were submitted to an auditory memory test (performed as described in 5_AFC memory test in adulthood).

*9 and 10_Fear extinction in adulthood*

Fear extinction was conducted in Context A and lasted 8 minutes and 40 seconds in total during which the first silent 160 seconds were followed by 20-seconds tone periods (same tone as in training) alternated with 40-seconds no-tone periods (the tone was presented 6 times during the extinction session). Two extinction sessions (approximately 3 hours interval between sessions) were performed the same day (age P86). During extinction no shock was delivered.

*11_CFC memory test postextinction*

Twenty four hours after extinction (age P87), a context memory test was performed as described in 4_CFC memory test in adulthood.

*12_AFC memory test postextinction.*

Two to four hours after CFC test at age P87, animals were submitted to an auditory memory test (performed as described in 5_AFC memory test in adulthood).

### 2.6. Plasma corticosterone measurement

Plasma corticosterone levels were measured after acute stress (forced swim). At the age of 4 months, rats were placed individually in cylinders filled with water and submitted to swimming stress for 5 minutes. Thirty minutes after the end of stress, rats were decapitated and their trunk blood collected in heparinized tubes. Plasma corticosterone levels were measured using an enzyme immunoassay kit (Correlate-EIA from Assay Designs Inc., USA).

### 2.7. Statistics

Freezing values along the 8 (CFC) or 5 minutes (AFC) of the test were evaluated with one-way ANOVA for repeated measurements. For comparison of rates, average duration and latencies at specific time points (continuous variables), unpaired Student's *t*-tests were applied. For comparison of number of freezing events (noncontinuous variables), the Mann-Whitney test was used. Statistics reported in the text and figures represent the mean ± SEM. Significance of results was accepted at *P* < .05. The SPSS statistical package was used to perform the analysis.

## 3. RESULTS

### 3.1. Stress prior to puberty does not have an immediate impact on basal anxiety and exploratory behavior: activity box, reactivity to novelty, and EPM

One week after the end of the stress period (i.e., at age P39-40) the basal anxiety and exploratory behavior of stressed and control animals were studied. The animals underwent three behavioral tests: EPM, activity box, and reactivity to novelty. There was no significant difference in any of the measured variables between stressed and control animals, neither for males nor for females (Table 1). For stressed animals, there was a significant sex difference in the time spent in the open arms of the EPM. Stressed females spent more time in the open arms than stressed males (effect not found for the control group). Percentage of time in the open arms: control males 13.93 ± 3.41 versus control females 18.83 ± 5.01 (n. s.); stressed males 14.4 ± 2.95 versus stressed females 24.15 ± 2.13 (*P* < .05). For control animals, there was a marginal sex effect in the time spent around the novel object. Control females spent more time around the novel object than control males (effect not found for the stressed group). Percentage of time around the object: control males 14.92 ± 3.43 versus control females 30.94 ± 7.73 (*P* = .053); stressed males 23.55 ± 4.97 versus stressed females 24.26 ± 2.13 (n. s.).

### 3.2. Differential impact of stress before puberty onthedevelopment of fear conditioning during adolescence

*1_AFC training in adolescence*

During the tone, stressed females showed significant lower percentages of time freezing and higher number of freezing events than control females and both groups of males (stressed and control). No differences were observed between stressed and control males. Percentage of time freezing: control males 38.35 ± 6.26 versus stressed males 47.93 ± 5.24 (n. s.); control females 48.01 ± 2.93 versus stressed females 31.28 ± 5.57 (*P* < .05). Number of freezing events: control males 3.11 ± 0.54 versus stressed males 3.70 ± 0.26 (n. s.); control females 4.83 ± 0.48 versus stressed females 3.33 ± 0.33 (*P* < .05).

*2_CFC test in adolescence*

**Figure 2:**
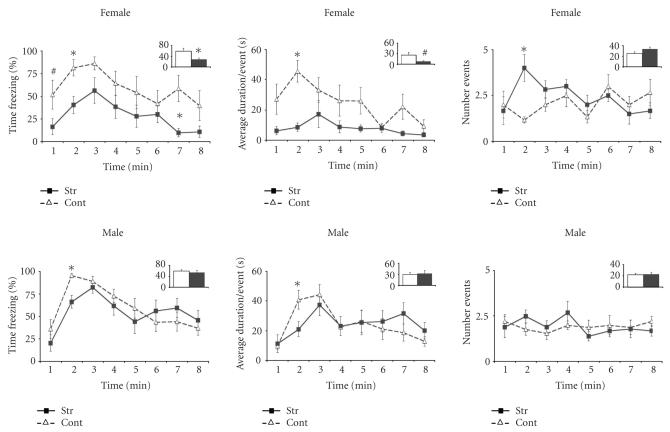
Contextual fear conditioning in adolescence. Mean percent freezing (left), average duration of freezing events (middle), and number of freezing events (right) (± SEM) on successive 1minute blocks during reexposure to the conditioned context in female (upper panel) and male (lower panel) rats that had been submitted to stress prior to the onset of puberty (filled squares) or control animals (open triangles). Within these figures, the total freezing value (percentage, average duration, or number) during the entire test is represented as bars in the inserts. *P < .05, ^#^ *P* < .05 < .10.

Stressed females displayed significantly lower levels of freezing than controls during the entire test (Figure 2 upper panel). This decrease in percentage of time freezing in stressed female rats resulted froman almost significant shortening of freezing events duration (*P* = .057), combined with an increase on the number of freezing events (n. s.) (see Figure 2 upper panel). No significant differences were observed in the freezing behavior between stressed and control males (see Figure 2 lower panel). There was a tendency towards a sex- and treatment-specific effect with control females behaving similarly to stressed and control males (F(1, 27) 3.406 P = .076; see Figure 2).

*3_AFC test in adolescence*

**Figure 3:**
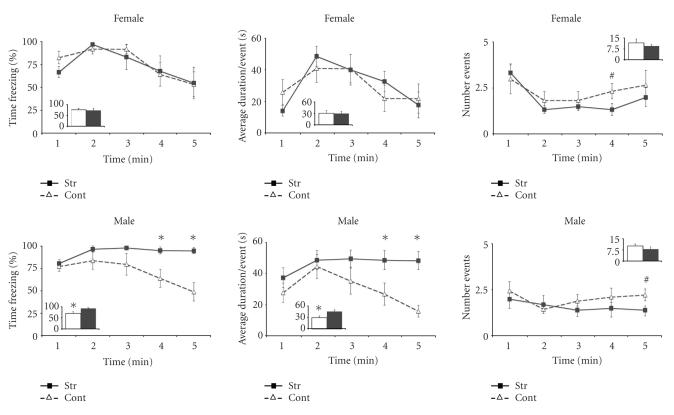
Auditory fear conditioning in adolescence. Mean percent freezing (left), duration of the average freezing event (middle), and number of freezing events (right) (± SEM) on successive 1minute blocks during reexposure to the conditioned tone in female (upper panel) andmale (lower panel) rats that had been submitted to stress prior to the onset of puberty (filled squares) or control animals (open triangles). Within these figures, the total freezing value (percentage, average duration, or number) during the entire duration of the tone is represented as bars in the inserts. *P < .05, ^#^ *P* < .05 < .10.

Stressed and control female rats showed similar freezing responses to the tone (see Figure 3 upper panel). Stressed males displayed significantly higher freezing levels than controls during the last half of the test (see Figure 3 lower panel). While stressed males spent virtually the entire 5 minutes of the auditory test freezing, control males and both female groups (stressed and control) showed a decrease in the time they spent freezing the longer the tone was on (i.e., they showed a ‘normal’ extinction to the tone; see Figure 3). (Percentage of time freezing during the second part of the tone: control males 61.09 ± 8.89 versus stressed males 95.61 ± 3.15 (*P* < .01); control females 65.25 ± 10.99 versus stressed females 65.61 ± 15.60 (n. s.)) (time × treatment interaction (F(2.489, 67.2) 3.247 *P* < .05; see Figure 3). The increase of the freezing time of stressed males resulted from a significant increase of the duration of the freezing events (*P* < .05) with no significant change of number of freezing events (n. s.) (see Figure 3 lower panel). When the behavioral responses during the 5 minutes tone delivery period were analyzed, stressed males showed higher percent of freezing levels than controls (*P* < .05), while female groups did not differ from each other (n. s.) (see Figure 3).

### 3.3. Impact of stress before puberty on fear conditioning during early adulthood

*4_CFC memory test in adulthood and 5_AFC memory test in adulthood.*

There were no significant differences in the freezing behavior of stressed and control animals when tested for their remote contextual and auditory fear memories in early adulthood (data not shown).

*6_AFC retraining in adulthood*

Stressed females spent significantly less time freezing during the tones. Control females behaved similarly to stressed and control males. Percentage of time freezing: control males 63.09 ± 6.54 versus stressed males 68.65 ± 5.79 (n. s.) control females 69.47 ± 6.21 versus stressed females 40.80 ± 10.62 (*P* < .05).

*7_CFC memory test after retraining in adulthood and 8_AFC memory test after retraining in adulthood*

There were no significant differences in the freezing behavior between stressed and control animals after retraining neither in the contextual nor in the auditory test. However, all groups spent less time freezing than before retraining (overall drop in percentage of time freezing).

*11_CFC memory test postextinction*

There were no significant differences in the freezing behavior of stressed and control animals in the contextual test after extinction (data not shown).

*12_AFC memory test postextinction*

**Figure 4:**
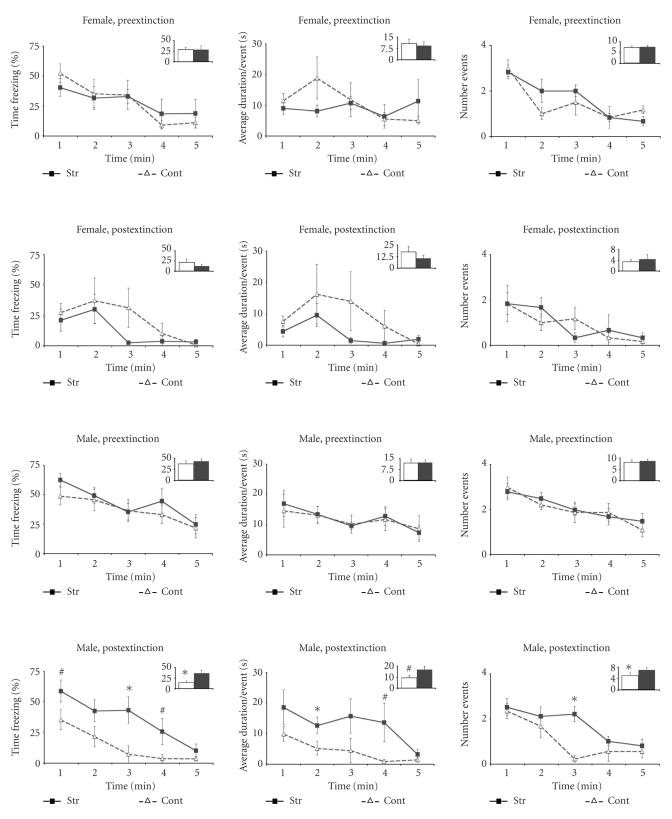
Auditory fear conditioning tests in adulthood before and after extinction. Mean percent freezing (left), duration of the average freezing event (middle), and number of freezing events (right) (± SEM) on successive 1minute blocks during reexposure to the conditioning tone in female (two upper panels) andmale (two lower panels) rats that were submitted to stress prior to the onset of puberty (filled squares) or control animals (open triangles).Within each panel the upper row shows the values before extinction and the lower row shows the values after extinction. Within these figures, the total freezing value (percentage, average duration, or number) during the entire duration of the tone is represented as bars in the inserts. *P < .05, ^#^ *P* < .05 < .10.

Stressed males displayed significantly higher levels of freezing than controls during the auditory test after extinction. That is, the percentage of time freezing and number of freezing events was significantly higher in stressed males than control males (compare in Figure 4 the two lower panels). The difference was significant in both halves of the tone test (first half: control males 24.33 ± 6.07 versus stressed males 49.32 ± 7.25 (*P* < .05); second half: control males 4.40 ± 1.94 versus stressed males 23.12 ± 8.04 (*P* < .05)). The effect was sex- and treatment-specific [F(1, 27) 6.171, *P* < .05]. Additional planned comparison to examined the specific effects of the extinction sessions showed that (a) stressed males maintained similar levels of freezing behavior during the auditory tests before and after extinction; while (b) control males showed a decrease in their levels of freezing behavior after extinction (compare in Figure 4 the two lower panels).

### 3.4. Stress before puberty did not have a significant impact on corticosterone levels 30 minutes after acute swim stress in adulthood.

There were no significant differences between stressed and control animals on the adult corticosterone plasma levels 30 minutes after an acute stress (5 minutes of forced swim). Plasma corticosterone concentrations (ng/mL): control males 329.64 ± 50.35 versus stressed males 364.17 ± 55.52 (n. s.); control females 301.77 ± 50.85 versus stressed females 293.65 ± 56.23 (n. s.).

## 4. DISCUSSION

We have studied (a) the effects of brief (3 days) psychogenic stress experienced before puberty on the acquisition, maintenance, and extinction of fear memories during adolescence and early adulthood in the rat; and (b) whether these effects are sex specific. We found that while animals from either sex were submitted to the same stressors and at exactly the same age, stressed male and female rats showed significant differences in the encoding, consolidation and expression of fear memories, which also differed from their respective nonstress controls. We also found that contextual and auditory fear memories were differentially affected by stress prior to puberty.

### 4.1. Sex-specific impact of stress before puberty on contextual and auditory fear memories measured during adolescence

Stress prior to puberty exerted opposite effects on the freezing behavior displayed by male and female rats (compared to their respective controls) during the contextual and auditory tests performed in adolescence. In females, stress before puberty induced a reduction of freezing to the context, but did not affect their behavior in the auditory test. Conversely, in males, stress before puberty did not affect freezing to the context, but increased the freezing responses to the conditioned tone.

Stress before puberty disrupted the expression of conditioned fear to the context in females while having a much weaker effect on males. This finding is consistent with, and extends, works using differentmodels of early life stress, such as social isolation given from weeks 3 to 13 [38] or separation from the mother for one hour per day from postnatal days 2 to 9 [39]. In both cases, fear conditioning was evaluated in adulthood and only stressed females showed a decrease in freezing during the contextual test.

A reduction in freezing in the fear conditioning test is conventionally interpreted as a reduction of fear memories. Different laboratories have found that stress enhances learning and memory processes in female rats. Hodes and Shors [26] showed that acute stress during adolescence enhanced the eyeblink conditioned responses in both males and female rats. Chronic stress during adulthood improved memory of female rats in a variety of spatial learning paradigms (such as the radial arm maze [31], object placement [32], the Y maze [33], and the Morris water maze [34]). The above-mentioned results may seem to contradict our finding of reduced freezing to the context in stressed females if we consider freezing as a measure of memory. Nevertheless, it remains to be established if the lower contextual freezing we observed in stressed female rats reflects a learning deficiency or it is rather the consequence of a change in the behavioral pattern of the response to fear (i.e., freeze versus flight) induced by stress. In support of the latter possibility, stressed female rats showed a hyper-reactive behavioral pattern during context testing: they froze more frequently but for shorter periods than the other experimental groups, and alternated more frequently from freezing to grooming and rearing behaviors. In any case, we should take into account the existence of important methodological differences between our study and the ones mentioned above, such as the type, intensity and duration of stressors, their timing with regards to animals' age, and the emotionality associated with the memory.

The sex-related impact of stress before puberty on auditory fear memories was opposite to that of contextual fear memories. That is, stress before puberty enhanced the expression of conditioned fear to the tone in males leading to virtually nonstop freezing during the entire 5 minutes of the tone during adolescence, while the other three groups showed a decay in the percentage of freezing time during the second half of the test. These findings differ from the ones obtained using other models of early life stress. Social isolation from weeks 3 to 13 [38] and separation from the mother for one or three hours from postnatal days 2 to 9 [39, 40] had no effect on the freezing response of males to AFC. Fifteen minutes handling from days 1 to 21 [40] or 24 hours maternal separation at postnatal day 4 [41] decreased the AFC freezing. Two facts may explain the disparity between the above-mentioned findings and ours. First, the animal's age during the fear conditioning training and test. While we found enhancement of conditioning freezing to the auditory test in adolescence, the studies mentioned above performed the fear conditioning training and test in adulthood. Indeed, when we performed AFC memory test in adulthood we did not find any difference in the freezing behavior between stress and control males. Second, the length of the stress period. For example, Luine and colleagues showed that for adult male rats the effects of stress upon certain types of memory differ for stress regimes of different lengths [28]. That is, in the radial arm maze, 1 week of restrain stress has no effect and 2 weeks improve learning and memory [42, 43], while 3 weeks of repeated restrain result in impaired memory [42, 43].

Differential effects of stress on contextual and auditory fear memories have also been reported by several groups. A single stress session facilitates subsequent contextual fear conditioning of adult male rats without affecting their auditory fear conditioning [44]. Isolation for some hours after fear conditioning reduced freezing to the context but not to the tone [45]. Rudy and colleagues showed that, at the molecular level, this dissociation may be mediated by glucocorticoids since (a) both adrenalectomy [46] or chronic exposure to high levels of the adrenal steroid dehydroepiandrosterone (DHEA, shown to have antiglucocorticoid properties) [47] impaired contextual fear conditioning but did not affect auditory fear conditioning; (b) injection of a glucocorticoid receptor antagonist either 1 hour prior to conditioning or immediately after conditioning reduced subsequent freezing to the context, without affecting freezing to the tone [48]. This dissociation may also take place at the anatomical level as contextual and auditory fear memories are encoded by different brain structures, with the amygdala being involved in both types of memories and the hippocampus mainly in memory for the context [49, 50]. In this line of thought, Calandreau and colleagues have recently shown that different amygdala nuclei are differentially involved in the two fear conditioning modalities [51]. Using a fear conditioning protocol similar to ours (shock paired to the tone), they showed that temporal inactivation of the basolateral amygdala (BLA) during training reduced freezing to the context, while inactivation of the lateral amygdala (LA) during training lead to decreased freezing exclusively during the second part of the auditory test. Taken together, these and our findings, we may speculate that stress before puberty might exert a differential impact in those two amygdala nuclei in males and females (i.e., the BLAmight become underactivated in females, while the LA hyperactivated in males). Stress prior to puberty may give rise to these (and other) dysfunctional activation patters by disrupting the developmental changes taking place in the brain during adolescence. For example, during this life period, the amygdala undergoes major synapse overproduction and pruning [52], while the hippocampus looses one quarter of its NMDA receptors on pyramidal regions [53]. It is noteworthy a recent study showing that maternal separation from postnatal days 2 to 20 significantly decreases the expression of synaptophysin (and therefore the number of synapses) in the hippocampus and amygdala (measured in 60-days old rats) [52].

### 4.2. Males stressed prior to puberty show a deficit to extinguish auditory fear memories in adulthood

One of the key findings of this work is that stress before puberty impaired the extinction of auditory fear memories during adulthood specifically in males. This finding is consistent with a previous study reporting an impairment to extinguish auditory fear memories in congenitally helpless rats [54]. The congenitally helpless rat strain was developed by selectively breeding for susceptibility to fail to escape foot shocks (in the learned helplessness/shuttle box paradigm) until 95% of the offspring showed spontaneously helpless behavior [55]. Shumake and colleagues found that congenitally helpless rats show abnormal metabolism in the prefrontal cortex [56] and reduced connectivity between forebrain and brainstem regions [57]. Based on the similarity of Shumake's and our findings, we could hypothesize that, in our study, stress prior to puberty results on abnormal functioning of the prefrontal cortex due to a disruption of the developmental changes taking place during adolescence. Abnormalities in the functioning of the prefrontal cortex may lead to the impairment to extinguish fear memories later in life found in our study. In agreement with this hypothesis, there is a substantial literature indicating that adolescence is a period characterized by a prominent developmental remodeling of the prefrontal cortex (a) volume reduction [58]; (b) loss of NMDA receptors [53] paralleled by an increase in the sensitivity induced by NMDA receptor antagonist [59]; (c) increase in the dopamine (DA) input (increase of DA fiber density [60], DA concentrations [61], and DA transporters [62]); and (d) increase in the cholinergic innervation [63]. Additionally, during adolescence there is an increase of the projections from the basolateral amygdala to the anterior cingulate cortex and the infralimbic subdivisions of medial prefrontal cortex [14]. These changes in the prefrontal cortex together with the above mentioned changes of the limbic brain and the connectivity between the prefrontal cortex and the amygdala lead to a shift in the balance between mesocortical and mesolimbic dopaminergic systems [15]. It should be noted that our auditory fear conditioning protocol differs from conventional studies in that extinction for auditory fear memories took place in a context different to the extinction context. Sustained fear responses in prepuberty stressed males might therefore also reflect an overgeneralization of the threatening cues in these animals to other contexts.

Our finding that stress prior to puberty has no impact on adult corticosterone levels 30 minutes after an acute stress is in agreement with previous reports showing that neither unpredictable chronic stress nor psychogenic stress during adolescence affected corticosterone release in adult rats [20, 22, 23].

Our stress protocol was based on the one developed in male rats by the group of Richter-Levin [17], in spite of including different stressors. These authors showed that prepubertal stress altered the behavioral consequences to a second stress in adulthood: it increased the susceptibility to develop learned helplessness in the shuttle box paradigm [18], while facilitating performance in the Morris water maze [17]. These learned helplessness and memory facilitation effects of prepubertal stress are in agreement with our results indicating increases freezing responses in the AFC for stressed males. Richter-Levin and colleagues have also shown enhanced basal anxiety when prepuberty stressed male rats are tested in adulthood [17–19]. Although we did not find significant differences in basal anxiety between control and stressed rats, it should be noted that, in difference to those studies, our work was performed in adolescent, not adult rats. Finally, when interpreting our results, we must take into consideration that the design of our study does not permit to conclude a differential susceptibility of adolescent rats to stress relative to adults since no adult-stressed group was included.

In summary, our findings indicate that stress before puberty onset affects the coping responses to emotional memories displayed by rats in adolescence and adulthood in a sex specific manner. To our knowledge, this is the first study that (a) investigates fear conditioning responses of animals stressed before puberty onset; and (b) evaluates the sexspecific impact of stress prior to puberty.

## Figures and Tables

**Table 1:** Effects of stress prior to puberty in the activity (open field and reactivity to novelty) and anxiety-like responses in the elevated plus maze (EPM) displayed by male and female rats during adolescence (P39-40). (Results are the mean ± SEM of *n* = 10 to 6 animals per group.)

	Males			Females		
	Control	Stressed	*P*	Control	Stressed	*P*

Open field, time in center (%)	16.93 ± 3.42	13.57 ± 2.50	.433	18.25 ± 4.14	14.67 ± 3.72	.534
Novel object, time exploring (%)	2.59 ± 0.61	3.40 ± 0.61	.365	4.44 ± 0.95	3.61 ± 0.84	.525
EPM, time at open arms (%)	13.93 ± 3.41	14.40 ± 2.95	.917	18.83 ± 5.01	24.16 ± 0.61	.362

## References

[1] Steinberg L, Dahl R, Keating D, Kupfer D, Masten A, Pine D, Cicchetti D (2005). The study of developmental psychopathology in adolescence: integrating affective neuroscience with the study of context. *Handbook of Developmental Psychopathology*.

[2] Penza KM, Heim C, Nemeroff CB (2003). Neurobiological effects of childhood abuse: implications for the pathophysiology of depression and anxiety. *Archives of Women's Mental Health*.

[3] Kessler RC (2003). Epidemiology of women and depression. *Journal of Affective Disorders*.

[4] Farrington DP, Loeber R (2000). Epidemiology of juvenile violence. *Child and Adolescent Psychiatric Clinics of North America*.

[5] Giedd JN, Blumenthal J, Jeffries NO (1999). Brain development during childhood and adolescence: a longitudinal MRI study. *Nature Neuroscience*.

[6] Sowell ER, Thompson PM, Holmes CJ, Jernigan TL, Toga AW (1999). In vivo evidence for post-adolescent brain maturation in frontal and striatal regions. *Nature Neuroscience*.

[7] Hayward C, Sanborn K (2002). Puberty and the emergence of gender differences in psychopathology. *Journal of Adolescent Health*.

[8] Dahl RE (2004). Adolescent brain development: a period of vulnerabilities and opportunities. Keynote address. *Annals of the New York Academy of Sciences*.

[9] Agid O, Kohn Y, Lerer B (2000). Environmental stress and psychiatric illness. *Biomedicine and Pharmacotherapy*.

[10] Kessler RC, Magee WJ (1993). Childhood adversities and adult depression: basic patterns of association in a US national survey. *Psychological Medicine*.

[11] Heim C, Plotsky PM, Nemeroff CB (2004). Importance of studying the contributions of early adverse experience to neurobiological findings in depression. *Neuropsychopharmacology*.

[12] McCormick CM, Mathews IZ (2007). HPA function in adolescence: role of sex hormones in its regulation and the enduring consequences of exposure to stressors. *Pharmacology Biochemistry and Behavior*.

[13] Fernández-Fernández R, Navarro VM, Barreiro ML (2005). Effects of chronic hyperghrelinemia on puberty onset and pregnancy outcome in the rat. *Endocrinology*.

[14] Cunningham MG, Bhattacharyya S, Benes FM (2002). Amygdalo-cortical sprouting continues into early adulthood: implications for the development of normal and abnormal function during adolescence. *Journal of Comparative Neurology*.

[15] Spear LP (2000). The adolescent brain and age-related behavioral manifestations. *Neuroscience and Biobehavioral Reviews*.

[16] Avital A, Ram E, Maayan R, Weizman A, Richter-Levin G (2006). Effects of early-life stress on behavior and neurosteroid levels in the rat hypothalamus and entorhinal cortex. *Brain Research Bulletin*.

[17] Avital A, Richter-Levin G (2005). Exposure to juvenile stress exacerbates the behavioural consequences of exposure to stress in the adult rat. *The International Journal of Neuropsychopharmacology*.

[18] Tsoory M, Cohen H, Richter-Levin G (2007). Juvenile stress induces a predisposition to either anxiety or depressive-like symptoms following stress in adulthood. *European Neuropsychopharmacology*.

[19] Tsoory M, Richter-Levin G (2006). Learning under stress in the adult rat is differentially affected by ‘juvenile’ or ‘adolescent’ stress. *The International Journal of Neuropsychopharmacology*.

[20] Maslova LN, Bulygina VV, Popova NK (2002). Immediate and long-lasting effects of chronic stress in the prepubertal age on the startle reflex. *Physiology and Behavior*.

[21] McCormick CM, Robarts D, Gleason E, Kelsey JE (2004). Stress during adolescence enhances locomotor sensitization to nicotine in adulthood in female, but not male, rats. *Hormones and Behavior*.

[22] McCormick CM, Robarts D, Kopeikina K, Kelsey JE (2005). Long-lasting, sex- and age-specific effects of social stressors on corticosterone responses to restraint and on locomotor responses to psychostimulants in rats. *Hormones and Behavior*.

[23] Isgor C, Kabbaj M, Akil H, Watson SJ (2004). Delayed effects of chronic variable stress during peripubertal-juvenile period on hippocampal morphology and on cognitive and stress axis functions in rats. *Hippocampus*.

[24] Sala-Catala J, Torrero C, Regalado M, Salas M, Ruiz-Marcos A (2005). Movements restriction and alterations of the number of spines distributed along the apical shafts of layer V pyramids in motor and primary sensory cortices of the peripubertal and adult rat. *Neuroscience*.

[25] Kabbaj M, Isgor C, Watson SJ, Akil H (2002). Stress during adolescence alters behavioral sensitization to amphetamine. *Neuroscience*.

[26] Hodes GE, Shors TJ (2005). Distinctive stress effects on learning during puberty. *Hormones and Behavior*.

[27] Bowman RE, Beck KD, Luine VN (2003). Chronic stress effects on memory: sex differences in performance and monoaminergic activity. *Hormones and Behavior*.

[28] Bowman RE (2005). Stress-induced changes in spatial memory are sexually differentiated and vary across the lifespan. *Journal of Neuroendocrinology*.

[29] Bowman RE, Maclusky NJ, Diaz SE, Zrull MC, Luine VN (2006). Aged rats: sex differences and responses to chronic stress. *Brain Research*.

[30] Shors TJ (2006). Stressful experience and learning across the lifespan. *Annual Review of Psychology*.

[31] Roof RL (1993). Neonatal exogenous testosterone modifies sex difference in radial arm and Morris water maze performance in prepubescent and adult rats. *Behavioural Brain Research*.

[32] Beck KD, Luine VN (2002). Sex differences in behavioral and neurochemical profiles after chronic stress: role of housing conditions. *Physiology and Behavior*.

[33] Conrad CD, Grote KA, Hobbs RJ, Ferayorni A (2003). Sex differences in spatial and non-spatial Y-maze performance after chronic stress. *Neurobiology of Learning and Memory*.

[34] Kitraki E, Kremmyda O, Youlatos D, Alexis MN, Kittas C (2004). Gender-dependent alterations in corticosteroid receptor status and spatial performance following 21 days of restraint stress. *Neuroscience*.

[35] Bowman RE, Zrull MC, Luine VN (2001). Chronic restraint stress enhances radial arm maze performance in female rats. *Brain Research*.

[36] Weiss EL, Longhurst JG, Mazure CM (1999). Childhood sexual abuse as a risk factor for depression in women: psychosocial and neurobiological correlates. *American Journal of Psychiatry*.

[37] Maren S (2001). Nuerobiology of Pavlovian fear conditioning. *Annual Review of Neuroscience*.

[38] Weiss IC, Pryce CR, Jongen-Rêlo AL, Nanz-Bahr NI, Feldon J (2004). Effect of social isolation on stress-related behavioural and neuroendocrine state in the rat. *Behavioural Brain Research*.

[39] Kosten TA, Miserendino MJD, Bombace JC, Lee HJ, Kim JJ (2005). Sex-selective effects of neonatal isolation on fear conditioning and foot shock sensitivity. *Behavioural Brain Research*.

[40] Kosten TA, Lee HJ, Kim JJ (2006). Early life stress impairs fear conditioning in adult male and female rats. *Brain Research*.

[41] Lehmann J, Pryce CR, Bettschen D, Feldon J (1999). The maternal separation paradigm and adult emotionality and cognition in male and female Wistar rats. *Pharmacology Biochemistry and Behavior*.

[42] Luine V, Martinez C, Villegas M, Magariños AM, McEwen BS (1996). Restraint stress reversibly enhances spatial memory performance. *Physiology and Behavior*.

[43] Luine V, Villegas M, Martinez C, McEwen BS (1994). Repeated stress causes reversible impairments of spatial memory performance. *Brain Research*.

[44] Cordero MI, Venero C, Kruyt ND, Sandi C (2003). Prior exposure to a single stress session facilitates subsequent contextual fear conditioning in rats: evidence for a role of corticosterone. *Hormones and Behavior*.

[45] Rudy JW, Kuwagama K, Pugh CR (1999). Isolation reduces contextual but not auditory-cue fear conditioning: a role for endogenous opioids. *Behavioral Neuroscience*.

[46] Pugh CR, Tremblay D, Fleshner M, Rudy JW (1997). A selective role for corticosterone in contextual-fear conditioning. *Behavioral Neuroscience*.

[47] Fleshner M, Pugh CR, Tremblay D, Rudy JW (1997). DHEA-S selectively impairs contextual-fear conditioning: support for the antiglucocorticoid hypothesis. *Behavioral Neuroscience*.

[48] Pugh CR, Fleshner M, Rudy JW (1997). Type II glucocorticoid receptor antagonists impair contextual but not auditory-cue fear conditioning in juvenile rats. *Neurobiology of Learning and Memory*.

[49] Kim JJ, Fanselow MS (1992). Modality-specific retrograde amnesia of fear. *Science*.

[50] LeDoux JE (1992). Brain mechanisms of emotion and emotional learning. *Current Opinion in Neurobiology*.

[51] Calandreau L, Desmedt A, Decorte L, Jaffard R (2005). A different recruitment of the lateral and basolateral amygdala promotes contextual or elemental conditioned association in Pavlovian fear conditioning. *Learning and Memory*.

[52] Andersen SL, Teicher MH (2004). Delayed effects of early stress on hippocampal development. *Neuropsychopharmacology*.

[53] Insel TR, Miller LP, Gelhard RE (1990). The ontogeny of excitatory amino acid receptors in rat
forebrain—I. *N*-*methyl*-*D*-*aspartate* and quisqualate receptors. *Neuroscience*.

[54] Shumake J, Barrett D, Gonzalez-Lima F (2005). Behavioral characteristics of rats predisposed to learned helplessness: reduced reward sensitivity, increased novelty seeking, and persistent fear memories. *Behavioural Brain Research*.

[55] Henn FA, Edwards E, Papolos DF, Lachman HM (1994). Animal models in the sutdy of genetic factors in human psychopathology. *Genetic Studies in Affective Disorders: Overview of Basic Methods, Current Directions and Critical Research Issues*.

[56] Shumake J, Poremba A, Edwards E, Gonzalez-Lima F (2000). Congenital helpless rats as a genetic model for cortex metabolism in depression. *NeuroReport*.

[57] Shumake J, Conejo-Jimenez N, Gonzalez-Pardo H, Gonzalez-Lima F (2004). Brain differences in newborn rats predisposed to helpless and depressive behavior. *Brain Research*.

[58] van Eden CG, Kros JM, Uylings HBM, Uylings HBM, van Eden CG, de Bruin JPC, Corner MA, Feenstra MGP (1990). The development of the rat prefrontal cortex: its size and development of connections with thalamus, spinal cord and other cortical areas. *Progress in Brain Research, the Prefrontal Cortex: Its Structure, Function and Pathology*.

[59] Farber NB, Wozniak DF, Price MT (1995). Age-specific neurotoxicity in the rat associated with NMDA receptor blockade: potential relevance to schizophrenia?. *Biological Psychiatry*.

[60] Kalsbeek A, Voorn P, Buijs RM, Pool CW, Uylings HBM (1988). Development of the dopaminergic innervation in the prefrontal cortex of the rat. *Journal of Comparative Neurology*.

[61] Leslie CA, Robertson MW, Cutler AJ, Bennett JP (1991). Postnatal development of D_1_ dopamine receptors in the medial prefrontal cortex, striatum and nucleus accumbens of normal and neonatal 6-hydroxydopamine treated rats: a quantitative autoradiographic analysis. *Developmental Brain Research*.

[62] Coulter CL, Happe HK, Murrin LC (1996). Postnatal development of the dopamine transporter: a quantitative autoradiographic study. *Developmental Brain Research*.

[63] Gould E, Woolf NJ, Butcher LL (1991). Postnatal development of cholinergic neurons in the rat: I. Forebrain. *Brain Research Bulletin*.

